# Baseline knowledge on chronic pulmonary aspergillosis and tuberculosis among health care workers involved in tuberculosis care in Uganda

**DOI:** 10.1016/j.ijregi.2025.100603

**Published:** 2025-02-19

**Authors:** Felix Bongomin, Ritah Nantale, Joseph Baruch Baluku, Tobius Odongo, Shamim Katusabe, Nixson Oyoo, Martin Muddu, Norman van Rhijn, David W Denning

**Affiliations:** 1 Manchester Fungal Infection Group, Division of Evolution, Infection and Genomics, Faculty of Biology, Medicine and Health, University of Manchester, Manchester, United Kingdom; 2 Department of Medical Microbiology and Immunology, Faculty of Medicine, Gulu University, Gulu, Uganda; 3 Department of Internal Medicine, Gulu Regional Referral Hospital, Gulu, Uganda; 4 Department of Community and Public Health, Faculty of Health Sciences Mbale, Busitema University, Mbale, Uganda; 5 Division of Pulmonology, Kiruddu National Referral Hospital, Kampala, Uganda; 6 Kitgum General Hospital, Kitgum, Uganda; 7 Makerere University Joint AIDS Program, Kampala, Uganda

**Keywords:** Chronic pulmonary aspergillosis, Pulmonary tuberculosis, Finger prick

## Abstract

**Objectives:**

Chronic pulmonary aspergillosis (CPA) often mimics pulmonary tuberculosis (PTB), leading to misdiagnosis and inappropriate management. We aimed to determine the baseline knowledge, practices, and self-rated confidence of health care workers (HCWs) regarding CPA in Uganda.

**Methods:**

Between January and April 2024, we conducted a cross-sectional survey involving HCWs at four public health facilities in Uganda currently enrolling patients with current or previously treated for PTB into a cohort study aimed at establishing the incidence of CPA. Data on sociodemographic characteristics, awareness, knowledge, practices regarding CPA, PTB, and finger-prick tests for point of care testing were collected using semi-structured questionnaires. Adequate knowledge was defined as a score of ≥80%.

**Results:**

A total of 110 HCWs participated, predominantly males (61, 57.5%), with a mean age of 38.8 years. Although most HCWs had heard of aspergillosis (78, 82.1%), only 23 (21.7%) demonstrated adequate knowledge about CPA. In addition, 25 (28.7%) reported encountering a patient with CPA in the past year, with 41 (47.1%) never routinely screening for CPA. Confidence in managing CPA was low among HCWs, with 29 (29.8%) expressing no confidence. Furthermore, only 22 (20.8%) had adequate knowledge about PTB. Referral hospital practice was significantly associated with higher odds of adequate CPA knowledge (adjusted odds ratio: 6.69, 95% confidence interval: 1.67-26.82, *P* = 0.007).

**Conclusions:**

There are significant gaps in awareness, knowledge, and practices of HCWs in Uganda related to CPA and PTB. Strategies to enhance training, guideline dissemination, and diagnostic capacity are essential to improve the management of these respiratory conditions and reduce misdiagnosis.

## Highlights

• Only 21.7% of health care workers (HCWs) had adequate knowledge of chronic pulmonary aspergillosis (CPA).
• Nearly half (47.1%) of HCWs did not routinely screen for CPA.
• Confidence in managing CPA was low, with 29.8% having no confidence.
• Referral hospital practice increased the likelihood of adequate CPA knowledge.
• Only 20.8% of HCWs had adequate knowledge of pulmonary tuberculosis.

## Introduction

Chronic pulmonary aspergillosis (CPA) is a spectrum of several progressive disease manifestations caused by the *Aspergillus* species in patients with underlying structural lung diseases, such as pulmonary tuberculosis (PTB), with 1- and 5-year mortality rates of about 5-30% and 40-60%, respectively [1]. CPA is categorized as simple aspergilloma (i.e. a fungal ball in a single, thin-walled cavity, without pericavitary infiltrates or pleural thickening), chronic cavitary pulmonary aspergillosis (i.e. one or more cavities with surrounding fibrosis, infiltrates, or pleural thickening), and chronic fibrosing pulmonary aspergillosis (i.e. fibrosis of two or more lobes with cavities) [2].

PTB-related CPA is an emerging global health problem, with an estimated annual incidence of about 1.8 million cases, resulting in 340,000 deaths [1]. PTB and CPA have similar clinical and radiologic manifestations [3,4]. CPA presents with chronic respiratory symptoms (i.e. cough, dyspnea, and hemoptysis) and systemic symptoms (i.e. fever, malaise, and weight loss), which are indistinguishable from those caused by PTB and other chronic respiratory diseases [3,4]. Radiologically, CPA manifests as consolidation, nodules, cavities, or progressive pleuroparenchymal fibrosis on imaging, which is also of striking resemblance with those of PTB and other cavitary lung diseases [3,4]. These similarities make the clinical suspicion and, therefore, the diagnosis of CPA complex in TB high-burdened countries.

An estimated 87,360 Ugandans develop PTB every year [5], resulting in an estimated 26,765 annual cases and 63,574 5-year period prevalent cases of CPA [6]. We have previously shown that the use of point of care *Aspergillus* immunoglobulin (Ig)G-IgM antibody testing improves diagnostic yields for CPA and contributes to anti-TB stewardship in Uganda [7]. However, there are no studies that have evaluated the knowledge and self-rated confidence in the diagnosis and treatment of CPA and TB diagnosis and management and finger-prick test practices for point-of-care diagnostics in health care workers (HCWs) in Uganda. We foresee in the near future that patients with unconfirmed and possibly recurrent PTB will be screened in TB clinics for CPA, using point-of-care tests using finger-prick blood samples as a part of routine clinical practice. Hence, this study was performed.

## Methods

### Study design and settings

We conducted a multi-centered, cross-sectional survey between January and April 2024. The study sites were Kiruddu National Referral Hospital in Kampala City, Gulu Regional Referral Hospital in Gulu City, Anaka General Hospital in Nwoya District, and Kitgum General Hospital in Kitgum District, all of which are in Uganda. These study sites are part of a large cohort study, the Chronic Pulmonary Aspergillosis: Optimization of Therapy, Immunogenetic Screening, and Diagnosis in Uganda (CPA-OPTIONS) study, a multi-center, prospective study, aimed at establishing the baseline prevalence and incidence of CPA in persons with PTB.

### Population

We enrolled HCWs (nurses, doctors, clinical officers with diploma in clinical medicine, pharmacists, laboratory scientists, and medical imaging technicians) involved in routine clinical care, including TB diagnosis and treatment. All participants were aged 18 years or older and provided informed consent to participate in the study.

### Data collection tool

The data collection tool was developed by FB and DWD, who are experts in CPA and infectious diseases, using CPA diagnosis and treatment guidelines for resource-limited settings [8]. A comparative set of questions on TB were developed using the World Health Organization TB guidelines [9] and on finger-prick testing using the World Health Organization's “fitness for purpose, real-time connectivity, ease of specimen collection, affordability, sensitivity, specificity, user-friendliness, rapidity and robustness, equipment-free operation, and deliverability”, acronymized as FIT-REASSURED criteria, for point-of-care tests [10,11].

### Outcomes

The primary outcome of this survey is knowledge and practice regarding CPA assessed using 12 sets of questions regarding awareness, risk factors, diagnosis, treatment, and complications of CPA. Secondary outcomes were knowledge and practices regarding TB and finger-prick testing.

### Data analysis

Data were analyzed in Stata version 15.0. Descriptive statistics such as frequency, percentages, mean, and SD were computed to summarize categorical and numerical data. The Bloom modified cut-off point was used to determine adequate CPA knowledge. This method classifies knowledge as adequate if the total score is ≥80% [12]. We conducted multivariable logistic regression to determine the factors associated with having adequate CPA knowledge. Variables with a *P* <0.2 at the bivariable analysis and those with scientific plausibility were added to the multivariable model. Significance level was set at *P* <0.05.

### Ethical considerations

The study protocol was approved by the Gulu University Research Ethics Committee (#GUREC-2023-717) and the University of Manchester University Research Ethics Committee 2 (Ref: 2024-20473-37733). All participants were aged 18 years or older and provided written informed consent. The institutional review boards of all study sites provided site approvals for the CPA_OPTIONS study and sub-studies.

## Results

### Sociodemographic characteristics of the study participants

A total of 110 participants were enrolled in this study, with a mean age of 34.8 ± 8.2 years. Of them, the majority (57.3%, n = 63) were aged less than 35 years, and 57.3% (n = 63) were male. Details are listed in Table 1.

**Table 1:** Socio-demographic characteristics of the study participants.

Characteristic	Frequency	Percentage
**Health facility**		
Gulu Regional Referral Hospital	28	25.4
Anaka General Hospital	25	22.7
Kitgum General Hospital	29	26.4
Kiruddu NRH	28	25.5
**Age of the participant (years), mean ±; 34.8±8.2 years**		
≤34	63	57.3
>34	47	42.7
**Sex of the participant**		
1. Male	63	57.3
2. Female	47	42.7
**Highest level of education**^a^		
1. Certificate	12	10.9
2. Diploma	37	33.6
3. Bachelors	55	50.0
4. Masters	6	5.5
**Designation**		
1. Nurse	39	35.4
2. Laboratory practitioners	15	13.6
3. Doctor	35	31.8
4. Clinical officer^b^	9	8.2
5. Others^c^	12	10.9
**Work experience, years, mean ± SD**	9.1 ± 7.2	

a Clinicians with a diploma in clinical medicine

b All levels are associated in medical fields including nursing, midwifery, laboratory technician, pharmacy, medicine and surgery, and many others

c Pharmacist, expert client, dispenser, medical imaging technologist, community linkage facilitator.

### Awareness and knowledge about chronic pulmonary aspergillosis

Most participants (82.7%, n = 91) had heard of aspergillosis; 81 (89.0%) knew that it is a fungal infection, 64 (70.3%) knew it is transmitted from the environment to humans, and 89 (97.8%) knew that the lungs are the organ most affected by aspergillosis. Overall, 25 (22.7%) participants had adequate knowledge about CPA, with a median knowledge score of 60.0% (40.0-73.3). A total of 27 (29.7%) participants knew that itraconazole was the recommended first-line treatment for CPA (Table 2) (Supplementary Table.

**Table 2:** Awareness and knowledge regarding chronic pulmonary aspergillosis in Uganda.

Variable	Frequency (n)	Percentage (%)
**Have you heard of the disease called aspergillosis?**		
Yes	91	82.7
**Questions assessing knowledge about chronic pulmonary aspergillosis (n = 91)**		
**What is aspergillosis?**		
1. A bacterial infection	8	8.8
2. A viral infection	1	1.1
3. A fungal infection	81	89.0
4. A parasitic infection	1	1.1
**How is aspergillosis transmitted?**		
1. Man to man	10	10.9
2. Animal to man	12	13.2
3. Environment to man	64	70.3
4. I don't know	5	5.5
**Which organ is most affected by aspergillosis?**		
1. Lungs	89	97.8
2. Skin	2	2.2
3. Brain	0	0.0
4. Kidney	0	0.0
**What are the risk factors for aspergillosis?**		
1. Smoking	2	2.2
2. Diabetes	1	1.1
3. Weakened immune system	31	34.0
4. All the above	57	62.6
**What are the symptoms of chronic pulmonary aspergillosis?**		
1. Fatigue	4	4.4
2. Persistent cough and hemoptysis	23	25.3
3. Weight loss	7	7.7
4. All of the above	68	74.7
**What diagnostic tests are used to diagnose chronic pulmonary aspergillosis? (Tick all that apply)**		
1. Blood culture	38	41.8
2. Chest X-ray	74	81.3
3. Sputum culture	61	67.0
4. Serum *Aspergillus*-specific immunoglobulin G levels	67	73.6
5. Bronchoalveolar lavage galactomannan	28	30.8
6. Beta D glucan	12	13.2
7. Skin prick test	18	19.8
8. I don't know	4	4.4
**What is the recommended first-line treatment for chronic pulmonary aspergillosis**		
1. Ketoconazole	1	1.1
2. Fluconazole	20	22.0
3. Itraconazole	27	29.7
4. Amphotericin B	27	29.7
5. I don't know	16	17.6
**What are the potential complications of chronic pulmonary aspergillosis? (Tick all that apply)**		
1. Respiratory failure	80	87.9
2. Massive bleeding leading to death	37	40.7
3. Lung cancer	26	28.6
4. Poor quality of life	46	50.6
5. I don't know	6	6.6
**How can chronic pulmonary aspergillosis be prevented?**		
1. Vaccination	6	6.6
2. Avoiding mold exposure	51	56.0
3. Early diagnosis and treatment	52	57.1
4. All of the above	15	16.5
5. I don't know	6	6.6

### Practices of HCWs regarding chronic pulmonary aspergillosis

A total of 27 (29.7%) participants had encountered a patient with CPA in the past year, whereas 42 (46.2%) had never encountered a CPA patient. In addition, 43 (47.3%) reported never routinely screening or testing patients with lung problems for CPA. Furthermore, 38 (41.8%) participants reported no experience with diagnosing CPA, and 22 (24.2%) had ever referred a patient with CPA to a specialist (Table 3).

**Table 3:** Practices of health care workers regarding chronic pulmonary aspergillosis in Uganda.

Variable	Frequency	Percentage
**Have you encountered any patients with chronic pulmonary aspergillosis in the past year?**		
1. Yes	27	29.7
**How often do you encounter patients with chronic pulmonary aspergillosis in your practice?**		
1. Frequently	2	2.2
2. Occasionally	10	11.0
3. Rarely	37	40.7
4. Never	42	46.2
**Do you routinely screen or test patients with lung problems for chronic pulmonary aspergillosis?**		
1. Often	6	6.6
2. Occasionally	12	13.2
3. Rarely	30	33.0
4. Never	43	47.3
**What is your usual approach to diagnosing chronic pulmonary aspergillosis?**		
1. No experience	38	41.8
2. Clinical symptoms assessment	38	41.8
3. Imaging studies	31	34.1
4. Laboratory tests	17	18.7
5. All of the above	3	3.3
**What is your usual approach to treating chronic pulmonary aspergillosis?**		
1. No experience	30	33.0
2. Antifungal medications	57	62.6
3. Antibiotics	14	15.4
4. Supportive care	28	30.8
5. All of the above	1	1.1
**Have you ever referred a patient with chronic pulmonary aspergillosis to a specialist?**		
1. Yes	22	24.2
2. No	66	75.9

### Self-rated confidence of HCWs regarding CPA management

**Figure 1:**
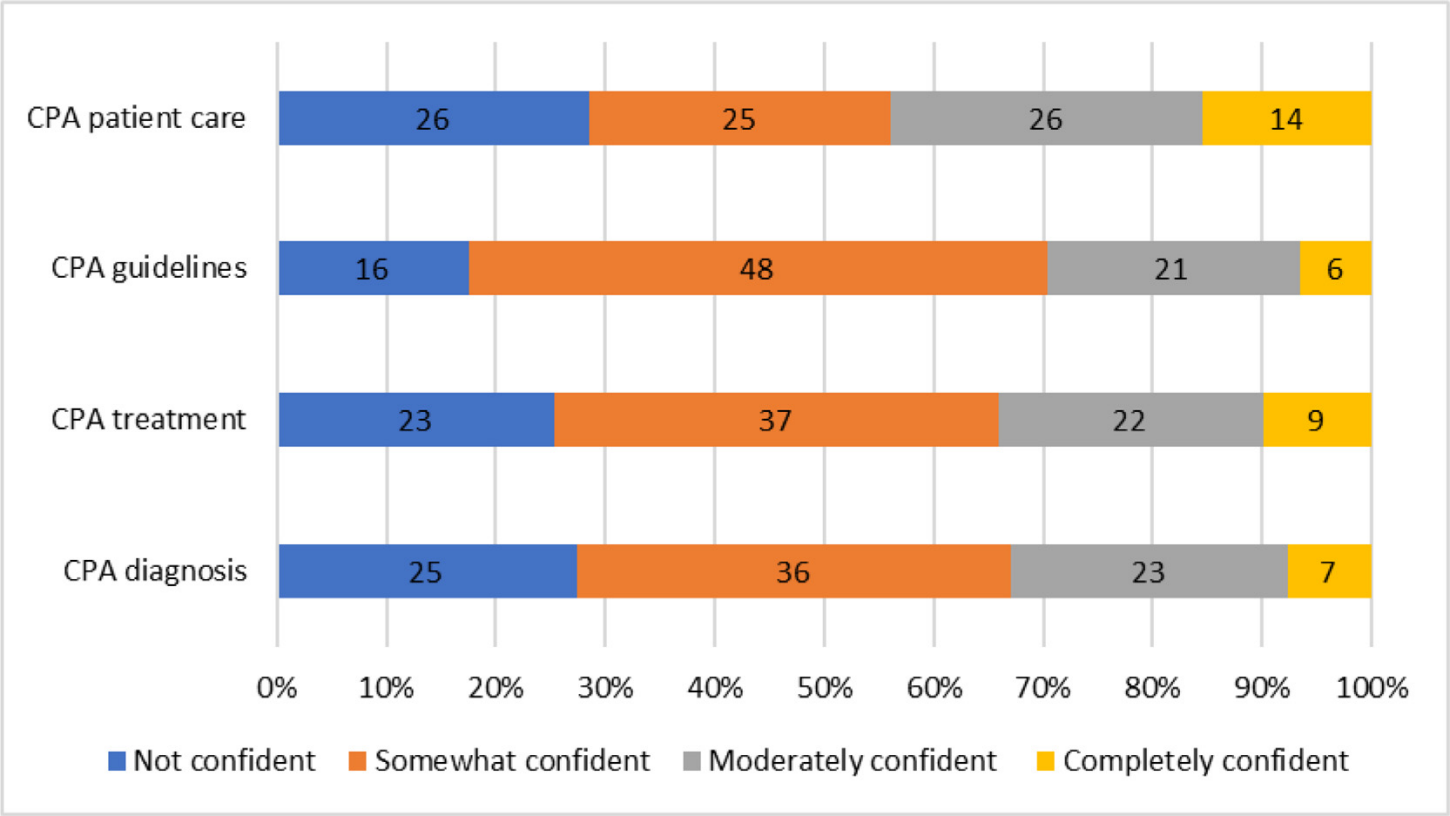
Self-rated confidence of health care workers regarding chronic pulmonary aspergillosis. CPA, chronic pulmonary aspergillosis.

Most of the HCWs reported feeling somewhat confident with the CPA guidelines (52.7%, n = 48), treatment (40.7%, n = 37), and diagnosis (39.5%, n = 36), respectively. Regarding providing care for patients with CPA, “completely confident” had the lowest frequency and the other categories shared similar levels (Figure 1).

### Predictors of adequate knowledge regarding CPA among HCWs in Uganda

HCWs practicing in referral hospitals had 7.69-fold higher odds of having adequate knowledge about CPA than those practicing in general hospitals (adjusted odds ratio: 7.66, 95% confidence interval: 1.90-30.84, *P* = 0.004). Other predictors had no significant impact on adequate knowledge (Table 4).

**Table 4:** Predictors of adequate knowledge regarding chronic pulmonary aspergillosis among health care workers in Uganda.

Variable	COR	95% CI	*P*-value	AOR	95% CI	*P*-value
Age in years						
≤34	1			1		
>34	0.44	(0.16-1.16)	0.095	0.61	(0.16-2.30)	0.462
Sex						
Male	1			1		
Female	0.44	(0.17-1.16)	0.095	0.42	(0.13-1.31)	0.134
Highest education level						
Certificate	1			1		
Diploma	0.16	(0.24-1.00)	0.050	0.22	(0.02-2.03)	0.181
Bachelors	0.45	(0.08-2.45)	0.354	0.31	(0.03-2.55)	0.274
Masters	-	-	-	-	-	-
Knowledge about tuberculosis						
Inadequate	1			1		
Adequate	2.39	(0.86-6.59)	0.094	2.56	(0.75-8.74)	0.134
Facility						
District hospital	1			1		
Referral hospital	7.50	(2.37-23.76)	0.001	7.66	(1.90-30.84)	**0.004**

AOR: adjusted odds ratio; CI: confidence interval; COR: crude odds ratio.

### Knowledge of HCWs about tuberculosis

A total of 22 (20.8%) participants declared adequate knowledge about TB. The mean knowledge score was 69.4 ± 15.8%. All participants knew that air droplets are the primary mode of transmission of PTB. Most (70.8%, n = 75) reported that latent TB infection is not contagious and 75.5% (n = 80) of the participants correctly identified the purpose of the Mantoux tuberculin skin test. A total of 56 (52.8%) participants did not know that bacillus Calmette-Guérin vaccination does not provide complete protection against TB. The majority (75.5%, n = 80) correctly stated that PTB and CPA have similar clinical and radiologic presentations (Table 5).

**Table 5:** Knowledge of health care workers about TB in Uganda.

Variable	Frequency (n)	Percentage (%)
**What is the primary mode of transmission of TB?**		
1. Airborne droplets	110	100.0
2. Foodborne	0	0.0
3. Bloodborne	0	0.0
4. Sexual contact	0	0.0
**Latent TB infection is contagious.**		
1. True	34	30.9
2. False	76	69.1
**What is the purpose of the Mantoux tuberculin skin test?**		
1. Diagnosis of active TB	26	23.6
3. Screening for latent TB infection	84	76.4
**Bacillus Calmette-Guérin vaccination provides almost complete protection against TB.**		
1. True	57	51.8
2. False	53	48.2
**What is the primary organ affected by TB?**		
1. Liver	1	0.9
2. Lungs	109	99.1
3. Kidneys	0	0.0
4. Heart	0	0.0
**How is active TB diagnosed definitively?**		
1. Chest X-ray	86	78.2
2. Sputum culture	97	88.2
3. Mantoux test	23	20.9
4. Urine lipoarabinomannan	75	68.2
5. Microscopy	82	74.6
**Pulmonary TB and chronic pulmonary aspergillosis have similar clinical and radiologic presentations.**		
1. True	84	76.4
2. False	26	23.6

TB, tuberculosis.

### Familiarity with tuberculosis treatment guidelines and drug-resistant tuberculosis among HCWs in Uganda

**Figure 2:**
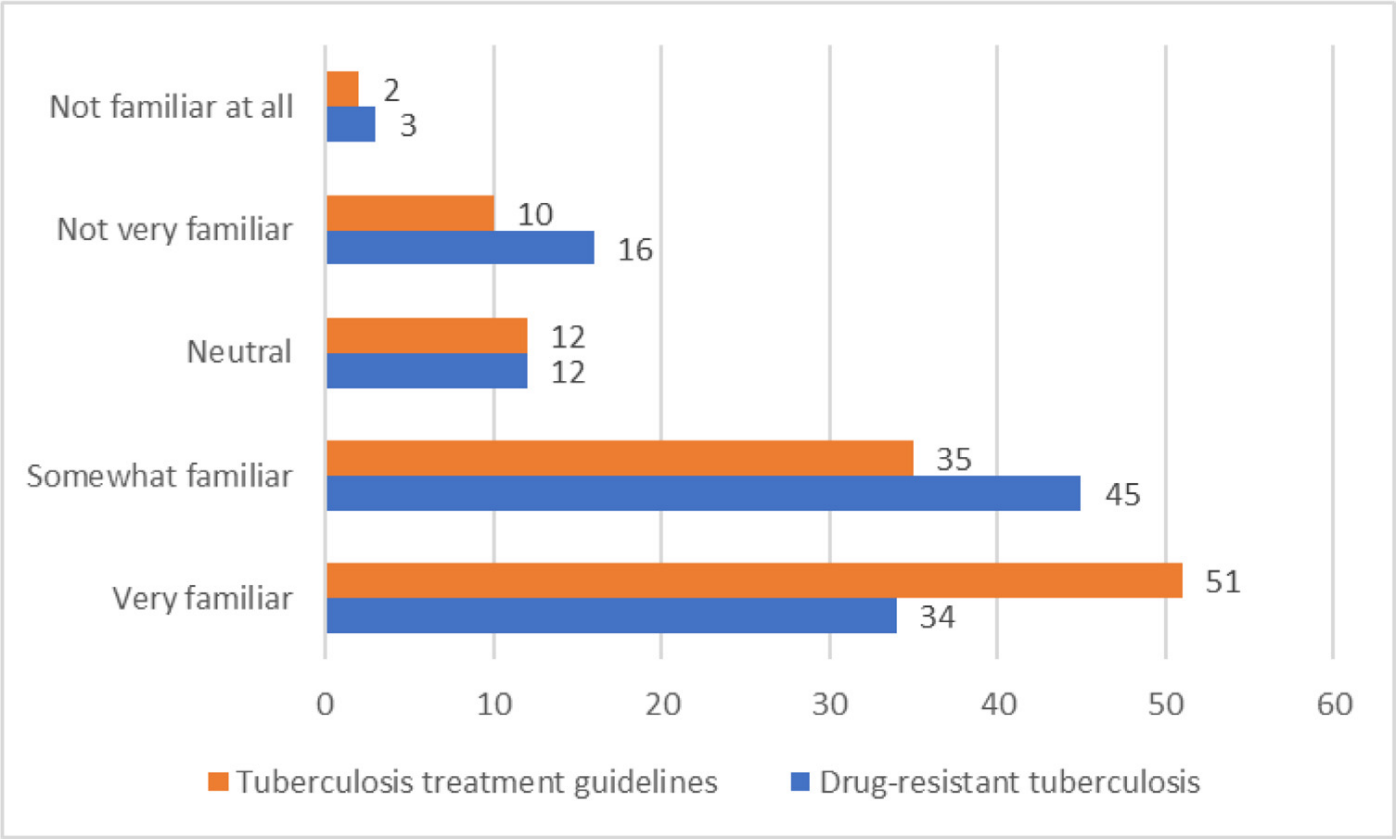
Familiarity with tuberculosis treatment guidelines and drug-resistant tuberculosis among health care workers in Uganda.

Most (46.4%, n = 51) participants reported that they were very familiar with TB treatment guidelines and 40.9% (n = 45) were somewhat familiar with drug-resistant TB (Figure 2).

### Knowledge and practice of finger-prick testing among HCWs in Uganda

A total of 64 (58.2%) had adequate knowledge about finger-prick testing for diagnosis. The mean knowledge score was 72.9 ± 14.8%. Most (71.8%, n = 79) participants had performed finger-prick testing at some time and 68.2% (n = 75) reported being completely confident with doing finger-prick testing. Regarding the potential barriers for implementing finger-prick testing, most cited limited test availability (69.1%, n = 76), lack of training (52.7%, n = 58), and patient discomfort (39.1%, n = 43), (Table 6).

**Table 6:** Knowledge and practice of finger-prick testing among health care workers in Uganda.

Variable	Frequency (n)	Percentage (%)
**Have you performed finger-prick testing in your practice?**		
1. Yes	79	71.8
2. No	31	28.2
**How confident are you in performing finger-prick test?**		
1. Not confident	5	4.6
2. Somewhat confident	8	7.3
3. Moderately confident	22	20.0
4. Completely confident	75	68.2
**What potential barrier do you see in implementing finger-prick testing in your health care setting?**		
1. Lack of training	58	52.7
2. Cost	25	22.7
3. Patient discomfort	43	39.1
4. The time it takes in clinic	8	7.3
5. Limited test availability	76	69.1
**Would finger-prick testing increase your workload in the clinic?**		
1. Yes, a lot	6	5.5
2. Yes, a little	18	16.4
3. Maybe	19	17.3
4. No	67	60.9
**Questions assessing knowledge about finger-prick testing**		
**What is the main advantage of finger-prick testing over traditional blood tests?**		
1. Faster results	80	72.7
2. Lower cost	27	24.6
3. Higher accuracy	2	1.8
4. Larger sample volume	1	0.9
**Finger-prick testing is only suitable for specific types of diseases and condition.**		
1. Yes	86	78.2
2. No	24	21.8
**Which diseases can be commonly diagnosed using finger-prick testing?**		
1. Diabetes mellitus	7	6.4
2. HIV	9	8.2
4. All of the above	94	85.5
**Finger-prick testing requires a specialized laboratory setup.**		
1. True	4	3.6
2. False	106	96.4
**In which health care scenarios do you think finger-prick testing is most beneficial?**		
1. Emergency situations	14	12.7
2. Routine checkups	4	3.6
3. Rural health care settings	9	8.2
4. All the above	83	75.5
**Finger-prick testing is primarily used for genetic testing.**		
1. True	11	10.0
2. False	99	90.0

## Discussion

CPA presents a significant challenge in clinical practice owing to its resemblance to PTB and the potential for misdiagnosis. Our study aimed to assess the baseline knowledge, practices, and self-rated confidence of HCWs regarding CPA in Uganda, with a focus on HCWs involved in a PTB cohort study. The findings shed light on several key areas, warranting attention and intervention to improve the management of respiratory conditions in Uganda. Subgroups of possible patients with PTB requiring screening for CPA and then management, if confirmed, are those with negative tests for PTB (clinically diagnosed or smear-negative) and those with apparent recurrence of PTB after cure because these patients have approximately 15-20% and >50% chance of CPA, respectively [13,14]. Co-infection of CPA with PTB and non-tuberculous mycobacterial disease are less frequent but do occur, as does the development of CPA during therapy for PTB.

The study revealed a notable gap in awareness and knowledge regarding CPA among HCWs. Although a majority had heard of aspergillosis, only a small proportion demonstrated adequate knowledge about CPA. This knowledge gap could contribute to under-recognition and mismanagement of CPA cases, emphasizing the urgent need for targeted educational initiatives to enhance HCWs’ understanding of this condition. Our study might also reflect the overall low (and the general public) knowledge levels of HCWs regarding fungal infections generally reported in several studies in low- and high-income settings, although a nuanced understanding of specific gaps is needed [[15], [16], [17]].

We found that HCWs practicing in referral hospitals had seven-fold higher odds of having adequate knowledge about CPA than those practicing in general hospitals. This is likely because they are practicing in an ecosystem that conducts periodic continuous medical education sessions and have higher cadre of physicians (consultants) who facilitate such training sessions. Our study, therefore, identifies HCWs at general hospitals (and, for that matter, low-level facilities) as a key demographic that should be targeted for CPA training.

The lack of enthusiasm for screening for CPA reported in our study could partly be because of the lack of diagnostics for CPA in Uganda [6]. Rapid screening options at present include chest x-ray (CXR) and/or serum *Aspergillus* IgG (with or without IgM) antibody assay. The LDBio lateral flow test is designed for use in the laboratory, although our recent work indicates that it can be used as a finger-prick assay as well, potentially in the clinic or at bedside [18,19]. Furthermore, the cost can be lowered by pooling samples, making it a very low-cost screening approach [18,19]. A normal CXR rules out CPA, and a positive *Aspergillus* IgG assay with a CXR showing pleural thickening, cavitation, or peri-cavitary infiltrates is highly suggestive of CPA [20].

Alternative and supplementary means of making and confirming a diagnosis of CPA are limited in Uganda. *Aspergillus* antibody tests are not available in the public sector, pulmonary computerized tomography scanning is seldom performed for CPA diagnosis, and bronchoscopy services are not readily accessible [6,21]. To support this, our respondents reported lack of the lateral flow assay as the most frequent barrier to diagnosing CPA, yet almost 70% reported to be completely confident in performing the test. Efforts should be put into availing CPA diagnostics in Uganda to foster early diagnosis and treatment. However, these low screening rates for fungal infections are not limited to low-resource settings. A nationwide study in the United States reported that only 3.7% health care providers reported frequently testing patients with pneumonia for coccidioidomycosis and 2.8% for histoplasmosis [22]. Thus, fungal infections continue to be neglected for diagnosis worldwide.

Regarding CPA treatment, only one in three respondents in our study identified itraconazole as the first-line treatment with another one in five suggesting fluconazole instead. This means that a good proportion of health workers in this setting might provide sub-optimal care for patients with CPA because fluconazole has no efficacy against aspergillosis [23]. Other studies have also identified anti-fungal therapy as a key knowledge gap among HCWs; a study among resident doctors in Nigeria revealed that more than 40% of respondents indicated fluconazole as a drug of choice in *Aspergillus* infection [24].

It was surprising to find low TB-related knowledge levels in our sample population considering the extensive TB infrastructure in the country and a dedicated national TB program that conducts health worker training. Moreover, individuals in our study were people partly or wholly involved in TB care. This is concerning and needs an extensive evaluation of HCWs’ TB knowledge levels and quality of TB services in Uganda. Nonetheless, expectedly, the mean TB knowledge scores were higher than CPA levels.

Our estimate of adequate levels of TB knowledge are significantly lower than what is reported among HCWs in Uganda by Buregyeya and colleagues [25] who reported 62% of HCWs to have adequate knowledge. This might be because they used a lower cutoff for determining adequate knowledge levels (70%) and sampled urban health workers. The trend of the association of adequate TB knowledge with CPA knowledge in our study indicates that training health workers on TB and CPA concurrent might have synergistic effect on HCW knowledge and practices.

## Limitations of the study

Despite its strengths, this study has several limitations that should be acknowledged. First, the cross-sectional nature of the survey limits our ability to establish causal relationships between variables. Although we identified factors associated with adequate knowledge about CPA among HCWs, longitudinal studies are needed to further explore the factors influencing knowledge acquisition and retention over time. Second, the study relied on self-reported data from HCWs, which may be subject to recall bias and social desirability bias. In addition, some questions assessing knowledge did not include an option of “I don't know.” This could potentially affect the accuracy and reliability of the reported knowledge and practices regarding CPA and TB. Future research could consider incorporating objective measures or validation studies to corroborate self-reported data. Lastly, the study was conducted at public health facilities in Uganda, which may limit the generalizability of the findings to other health care settings or regions. Further research in diverse settings and populations is needed to ensure the broader applicability of the study findings and capture variations in knowledge, practices, and confidence levels among HCWs.

### Areas for future implementation research

Future research should focus on evaluating the effectiveness of educational interventions and training programs aimed at improving HCWs’ knowledge, practices, and confidence regarding CPA and TB management. Longitudinal studies are needed to assess the impact of these interventions on clinical outcomes, patient satisfaction, and health care delivery in Uganda. In addition, implementation research is needed to identify barriers and facilitators to the adoption and implementation of evidence-based guidelines and diagnostic tools for respiratory conditions in Ugandan health care settings. This could involve qualitative studies to explore HCWs’ perspectives, organizational factors, and system-level challenges influencing the delivery of respiratory care. Moreover, there is a need for research to assess the cost-effectiveness of different strategies for improving respiratory disease management in Uganda, including the integration of CPA and TB services, optimization of diagnostic algorithms, and use of innovative technologies for screening and diagnosis. Such studies can inform resource allocation decisions and policy development aimed at strengthening respiratory health care systems in Uganda and similar settings.

## Conclusion

There are significant gaps in HCWs’ awareness, knowledge, and practices related to CPA and PTB in Uganda. The identified gaps in awareness, knowledge, and practices regarding CPA and TB highlight the need for targeted educational interventions and training programs for HCWs in Uganda. These initiatives should focus on improving diagnostic skills, enhancing awareness of guidelines, and addressing misconceptions about respiratory conditions. Future interventions should consider strategies to optimize institutional support, such as regular training programs, mentorship opportunities, and knowledge-sharing platforms, to enhance HCWs’ competency in managing respiratory conditions.

## Declarations of competing interest

The authors have no competing interests to declare.

## Appendix Supplementary materials

Image, application 1
