# Supplementary material for: Baseline knowledge on chronic pulmonary aspergillosis and tuberculosis among health care workers involved in tuberculosis care in Uganda
Source: IJID Reg. 2025 Feb 19;14:100603. doi: 10.1016/j.ijregi.2025.100603 (PMC11937940; doi:10.1016/j.ijregi.2025.100603)
Supplement: Supplementary file 1 [file mmc1.docx]

| **Questions assessing knowledge about chronic pulmonary aspergillosis** | |
| --- | --- |
| **What is aspergillosis?** |  |
| 1. A bacterial infection | Incorrect |
| 2. A viral infection | Incorrect |
| 3. A fungal infection | Correct |
| 4. A parasitic infection | Incorrect |
| **How is aspergillosis transmitted?** | |
| 1. Man to man. | Incorrect |
| 2. Animal to man | Incorrect |
| 3. Environment to man | Correct |
| 4. I don’t know. | Incorrect |
| **Which organ is most affected by aspergillosis?** | |
| 1. Lungs | Correct |
| 2. Skin | Incorrect |
| 3. Brain | Incorrect |
| 4. Kidneys | Incorrect |
| **What are the risk factors for aspergillosis?** | |
| 1. Smoking | Incorrect |
| 2. Diabetes | Incorrect |
| 3. Weakened immune system | Incorrect |
| 4. All the above | Correct |
| **What are the symptoms of chronic pulmonary aspergillosis?** | |
| 1. Fatigue | Incorrect |
| 2. Persistent cough and hemoptysis | Incorrect |
| 3. Weight loss | Incorrect |
| 4. All the above | Correct |
| **What diagnostic tests are used to diagnose chronic pulmonary aspergillosis? (Tick all that apply)** | |
| 1. Blood culture | Incorrect |
| 2. Chest X-ray | Correct |
| 3. Sputum culture | Correct |
| 4. Serum Aspergillus specific IgG levels | Correct |
| 5. Bronchoalveolar lavage galactomannan | Correct |
| 6. Beta D glucan | Incorrect |
| 7. Skin prick test | Incorrect |
| 8. I don’t know | Incorrect |
| **What is the recommended first-line treatment for chronic pulmonary aspergillosis** | |
| 1. Ketoconazole | Incorrect |
| 2. Fluconazole | Incorrect |
| 3. Itraconazole | Correct |
| 4. Amphotericin B | Incorrect |
| 5. I don’t know | Incorrect |
| **What are the potential complications of chronic pulmonary aspergillosis? (Tick all that apply)** | |
| 1. Respiratory failure | Correct |
| 2. Massive bleeding leading to death | Correct |
| 3. Lung cancer | Incorrect |
| 4. Poor quality of life | Correct |
| 5. I don’t know | Incorrect |
| **How can chronic pulmonary aspergillosis be prevented?** | |
| 1. Vaccination | Incorrect |
| 2. Avoiding mold exposure | Correct |
| 3. Early diagnosis and treatment | Correct |
| 4. All the above | Incorrect |
| 5. I don’t know | Incorrect |

| **Questions assessing knowledge about Tuberculosis** | |
| --- | --- |
| **What is the primary mode of transmission of tuberculosis?** | |
| 1. Airborne droplets | Correct |
| 2. Foodborne | Incorrect |
| 3. Bloodborne | Incorrect |
| 4. Sexual contact | Incorrect |
| **Latent tuberculosis infection is contagious.** | |
| 1. True | Incorrect |
| 2. False | Correct |
| **What is the** **purpose of the Mantoux tuberculin skin test?** | |
| 1. Diagnosis of active tuberculosis | Incorrect |
| 2. Identification of drug resistance | Incorrect |
| 3. Screening for latent tuberculosis infection | Correct |
| 4. Monitoring treatment response | Incorrect |
| **BCG vaccination provides almost complete protection against tuberculosis.** | |
| 1. True | Correct |
| 2. False | Incorrect |
| **What is the primary organ affected by tuberculosis?** | |
| 1. Liver | Incorrect |
| 2. Lungs | Correct |
| 3. Kidneys | Incorrect |
| 4. Heart | Incorrect |
| **How is active tuberculosis diagnosed definitively?** | |
| 1. Chest X-ray | Incorrect |
| 2. Sputum culture | Correct |
| 3. Mantoux test | Incorrect |
| 4. Urine lipoarabinomannan | Correct |
| 5. Microscopy | Correct |
| **Pulmonary tuberculosis and chronic pulmonary aspergillosis have similar clinical and radiological presentations** | |
| 1. True | Correct |
| 2. False | Incorrect |

| **Questions assessing knowledge about fingerprick testing** | |
| --- | --- |
| **What is the main advantage of fingerprick testing over traditional blood tests?** | |
| 1. Faster results | Correct |
| 2. Lower cost | Correct |
| 3. Higher accuracy | Incorrect |
| 4. Larger sample volume | Incorrect |
| **Fingerpick testing is only suitable for specific types of diseases and condition** | |
| 1. Yes | Correct |
| 2. No | Incorrect |
| **Which diseases can be commonly diagnosed using fingerprick testing?** | |
| 1. Diabetes mellitus | Incorrect |
| 2. HIV | Incorrect |
| 3. Hepatitis B | Incorrect |
| 4. All of the above | Correct |
| **Fingerpick testing requires a specialized laboratory setup** | |
| 1. True | Incorrect |
| 2. False | Correct |
| **In which healthcare scenarios do you think fingerpick testing is most beneficial** | |
| 1. Emergency situations | Incorrect |
| 2. Routine check-ups | Incorrect |
| 3. Rural healthcare settings | Incorrect |
| 4. All the above | Correct |
| **Fingerpick testing is primarily used for genetic testing.** | |
| 1. True | Incorrect |
| 2. False | Correct |
